# Willingness to use HIV prevention methods among vaccine efficacy trial participants in Soweto, South Africa: discretion is important

**DOI:** 10.1186/s12889-020-09785-0

**Published:** 2020-11-07

**Authors:** Fatima Laher, Taibat Salami, Stefanie Hornschuh, Lerato M. Makhale, Mamakiri Khunwane, Michele P. Andrasik, Glenda E. Gray, Hong Van Tieu, Janan J. Dietrich

**Affiliations:** 1 Perinatal HIV Research Unit, Faculty of Health Sciences, University of the Witwatersrand, Diepkloof, P.O. Box 114, Johannesburg, Soweto 1864 South Africa; 2 School of Medicine, University of Texas, San Antonio, TX USA; 3 Vaccine and Infectious Disease Division, Fred Hutchinson Cancer Research Center, Seattle, Washington USA; 4 South African Medical Research Council, Cape Town, South Africa; 5 Laboratory of Infectious Disease Prevention, New York Blood Center, New York, NY USA; 6 Division of Infectious Diseases, Columbia University Medical Center, New York, NY USA

**Keywords:** HIV, Prevention, Preferences, Vaccine, Injectable

## Abstract

**Background:**

Despite multiple available HIV prevention methods, the HIV epidemic continues to affect South Africa the most. We sought to understand willingness to use actual and hypothetical HIV prevention methods among participants enrolled in a preventative HIV vaccine efficacy trial in Soweto, South Africa.

**Methods:**

We conducted a qualitative study with 38 self-reporting HIV-uninfected and consenting 18–35 year olds participating in the HVTN 702 vaccine efficacy trial in Soweto. Using a semi-structured interview guide, five focus group discussions (FGDs) were held, stratified by age, gender and sexual orientation. The FGDs were composed of: (i) 10 heterosexual women aged 18–24 years; (ii) 9 heterosexual and bisexual women aged 25–35 years; (iii & iv) heterosexual men aged 25–35 years with 7 in both groups; and (v) 5 men aged 18–35 years who have sex with men. FGDs were audio-recorded, transcribed verbatim, translated into English and analysed using thematic analysis.

**Results:**

We present five main themes: (i) long-lasting methods are preferable; (ii) condoms are well-known but not preferred for use; (iii) administration route of HIV prevention method is a consideration for the user; (iv) ideal HIV prevention methods should blend into the lifestyle of the user; and the perception that (v) visible prevention methods indicate sexual indiscretion.

**Conclusions:**

The participants’ candour about barriers to condom and daily oral pre-exposure prophylaxis (PrEP) use, and expressed preferences for long-lasting, discreet, lifestyle-friendly methods reveal a gap in the biomedical prevention market aiming to reduce sexually acquired HIV in South Africa. Product developers should consider long-acting injectable formulations, such as vaccines, passive antibodies and chemoprophylaxis, for HIV prevention technologies. Future innovations in HIV prevention products may need to address the desire for the method to blend easily into lifestyles, such as food-medication formulations.

## Background

Individuals can choose from an array of strategies to prevent acquisition of Human Immunodeficiency Virus (HIV). Widely available strategies include: behavioural strategies such as risk reduction counselling and HIV testing [1] which aim to reduce risky behaviours; barrier methods such as male and female condoms which aim to reduce sexual transmission [2]; the surgical method of voluntary medical male circumcision (VMMC) which reduces biological risk of HIV acquisition for men [3]; and antiretroviral drug methods such as the prevention of mother-to-child transmission [4], treatment as prevention which aim to reduce transmission to partners [5], and post-exposure prophylaxis (PEP) [6]. Systemic (daily oral tablet) pre-exposure prophylaxis (PrEP) is becoming more widely accessible, local (vaginal ring) PrEP is an emerging strategy and long-acting injectable and subcutaneous implant PrEP formats are being trialled [7, 8]. Conditional cash transfers, which aim to address structural drivers of risk, may reduce risky behaviours in young women [9] but are not yet widespread policy. Strategies under research for HIV prevention include vaccines, passive antibodies and microbicides [10].

There are advantages and drawbacks of the different HIV prevention strategies. None of the current strategies offer complete protection against HIV or other sexually acquired conditions such as sexually transmitted infections (STIs) and pregnancy. Therefore, a combination intervention approach is recommended [11]. Even though counselling offers information on methods to reduce risk, individuals are still faced with the task of tailoring their own HIV prevention method mix based on locally available options, and there is limited understanding of how individuals navigate such choices [12]. Condoms and, in some regions, voluntary medical male circumcision [13] are popular and accessible prevention strategies. However, the former relies on adherence and, like many sexual behaviour change methods, is challenged by gender power dynamics [14], and the latter is a method that cannot always be controlled by women or men who have sex with men (MSM) who engage in receptive anal sex. Current licensed drug strategies of PEP and oral PrEP rely on daily adherence.

Despite multiple accessible prevention methods, HIV remains a critical public health concern. In 2017 alone, there was an estimated 1.8 million new infections worldwide: South Africa accounted for 15% of all new infections and 19% of the total global HIV-infected population. In South Africa, the epidemic is transmitted mostly heterosexually, and young women are a most-at-risk population [15, 16].

Given the continued HIV burden in South Africa, there is an evident need for novel strategies to add to the mix of prevention methods for at-risk individuals. The search for a preventative HIV vaccine continues. HVTN 702, a phase 2b/3 randomised, double-blind, placebo-controlled clinical trial, investigated the efficacy of ALVAC-HIV plus Bivalent Subtype C gp120/MF59 amongst at-risk HIV-uninfected young adults in South Africa [17]. In February 2020, HVTN 702 concluded that the vaccine regimen was not efficacious. Observed HIV incidence was high, regardless of vaccine or placebo receipt, and despite trial participants having been routinely offered standard HIV prevention methods including risk reduction counselling, VMMC, oral PrEP, PEP, barrier methods, and voluntary counselling and testing for HIV. Participants had been counselled at all visits to use prevention methods based on their personal preferences and circumstances. It is in this context of an HIV-1 preventative vaccine efficacy trial that our qualitative study assessed willingness to use different HIV prevention methods amongst trial participants.

## Methods

We conducted a qualitative study using five focus group discussion (FGDs) and a demographic questionnaire.

### Setting

Our qualitative study was conducted from September to October 2018, during the HVTN 702 trial, at the Perinatal HIV Research Unit (PHRU). The PHRU is located at the Chris Hani Baragwanath Academic Hospital in Soweto, southwest of Johannesburg, South Africa. The Soweto population was estimated to be 1,271,628 people and its population density 6357 persons/square kilometre [18]. In October 2016, PHRU was the first of 14 trial sites in South Africa to enrol participants into the HIV preventative vaccine efficacy trial HVTN 702, also known as “Uhambo” (“Journey”) [19]. To be enrolled into HVTN 702, participants had previously been assessed by the trial staff for 34 eligibility criteria: in summary, they had to be 18–35 years old, HIV-uninfected healthy at-risk adults, willing to receive HIV test results, discuss HIV risk and receive risk reduction counselling, and women were required to be on contraception and not pregnant or breastfeeding.

### Eligibility

Eligibility criteria for our qualitative study included enrolled PHRU HVTN 702 participants who stated they were currently HIV-uninfected, 18–35 years old, and could provide written voluntary informed consent. We excluded pregnant or breastfeeding women and those with social or psychiatric conditions. Each FGD was limited to 10 participants to allow interaction and multiple perspectives.

### Data collection

Using purposive sampling and a quota system, potential participants were recruited face-to-face over one month at the PHRU HVTN 702 clinical trial facility, and through paper flyers posted on PHRU noticeboards. The staff for this study, who were not involved in the HVTN 702 trial, explained this study to potential participants, and interested individuals completed a screening questionnaire in two parts (Table 1a). We scheduled individuals to the relevant FGD in chronological order of their recruitment, until a maximum of 10 individuals scheduled per FGD was reached. Scheduled individuals received reminders the night before their scheduled FGD. On FGD day, participants provided written consent, completed a demographic questionnaire, received a standardised information session about existing HIV prevention methods and administration routes, and thereafter, participated in the FGD.

**Table 1:** Summary of data collection process

a. Screening questionnaire	Data collected
*Participant self-report on electronic computer tablet at recruitment*	Name, phone numbers, age range, gender, sexual orientation, HIV status, length of participation in HVTN 702 study, HVTN study number
*Interviewer-administered by telephone calls with potentially eligible participants*	Willingness to participate in FGD, availability considerations for scheduling FGD
b. Demographic questionnaire	**Variables assessed**
*Participant self-report with pen and paper format questionnaire immediately prior to FGD*	Date of birth, gender, sexual orientation, marital status, highest level of education completed, average monthly household income, source of income in the last three months, housing type, HIV risk perception of self and of partner
c. Focus group discussion	**Topics**
*Semi-structured interview guide*	Overall HIV prevention knowledge, experience and perceptions about HIV vaccines, HIV counselling and testing, condoms, VMMC, antiretroviral medications, PrEP, hypothetical methods and ultimate preferences for prevention

### Demographic questionnaire

Each participant completed a demographic questionnaire in English (Table 1b). Multilingual study staff read the questionnaire, explaining in English and/or local languages as preferred.

### Focus group discussions

To achieve data triangulation and saturation, we conducted multiple FGDs with different participant strata. Trained interviewers conducted five FGDs stratified by age, gender and sexuality: (i) heterosexual women aged 18–24 years; (ii) heterosexual and bisexual women aged 25–35 years; (iii & iv) heterosexual men aged 25–35 years; and (v) men aged 18–35 years who have sex with men. With no new codes emerging in the final FGD, data saturation was achieved.

Interviewers used a semi-structured interview guide containing open-ended questions with probes (Table 1c). FGDs lasted 90–110 min and were conducted in a private room at the PHRU, away from the HVTN 702 clinic space. FGDs with women were led by one local multilingual female facilitator and one English-speaking young female facilitator. FGDs with men were led by one local multilingual male facilitator and one multilingual female facilitator. Interviewers were experienced in qualitative research and trained on HIV prevention strategies. FGDs were conducted in a mix of local languages (English, Zulu, and Sotho). FGDs were audio-recorded, transcribed verbatim and translated into English. Transcripts were validated with the audio-recording.

### Ethical and community considerations

Ethical approval was obtained from the University of the Witwatersrand Human Research Ethics Committee, the HVTN 702 protocol team and the PHRU HIV prevention community advisory board. Participants were provided lunch and reimbursed ZAR150 (~USD12) for transport costs.

### Data analysis

Quantitative data from the demographic questionnaire were entered into an online database on SurveyPlanet [20]. Descriptive statistics and frequencies were calculated using Microsoft Excel.

Qualitative data were analysed using thematic analysis through a priori coding framework. The first analyst read through the transcripts to gain an understanding of the data categories. The first analyst discussed the categories with the second analyst, and they agreed upon the following categories, taken from the FGD interview guide: HIV prevention methods preferences, experiences, barriers, facilitators, HIV vaccine, VMMC, antiretroviral, PrEP, condoms and hypothetical HIV prevention innovations. The second analyst categorised the data by assigning text with categories in a line-by-line analysis. Thereafter, the coding process involved open coding whereby the second analyst conducted a line-by-line analysis assigning codes to text. After the first two FGD transcripts had been coded, the second analyst discussed the initial codes with two researchers experienced in qualitative data analysis. Codes were agreed upon and refined, and a codebook was developed to guide the manual coding of all five transcripts. The second analyst then continued with the process of axial coding to understand the relationships between the codes. A third analyst co-coded the transcripts and regularly met with the second analyst for discussions to reach consensus. The primary analyst formulated themes from the codes and discussed with co-authors until consensus was reached.

## Results

### Demographics

Of 81 HVTN 702 participants approached, 71 individuals completed the pre-screening assessment, 68 were eligible and 38 participated in this qualitative study. The median age of participants was 26 years; 50% were men, 84% identified as heterosexual, 97% were single or widowed, 79% completed high school, 58% had a monthly household income between ZAR 1000–5000 (~USD83–400) and 79% lived in formal housing (Table 2). For perception of HIV acquisition risk, 53% reported high/moderate risk, and 39% reported that their partners were at high/ moderate risk.

**Table 2:** Demographic characteristics of the 38 focus group discussion participants

Demographic characteristic	
*Median age in years* (IQR)	26 (23–30)
*Gender,* n (%)	
Male	19 (50)
Female	18 (47)
Transgender	1 (3)
*Sexual Orientation,* n (%)	
Heterosexual	32 (84)
Men who have sex with men	5 (13)
Bisexual	1 (3)
*Marital Status,* n (%)	
Married (legal or traditional) or living as married	1 (3)
Single/widowed	37 (97)
*Highest education completed,* n (%)	
Undergraduate degree	5 (13)
High School	30 (79)
Primary school	1 (3)
No schooling	2 (5)
*Monthly household income,* n (%)	
Less than R1000	11 (29)
R1000 - R5000	22 (58)
R5000 - R10,000	4 (11)
More than R10,000	1 (3)
*Source of income in last 3 months,* n (%)*	
Job/self-employed	15 (39)
Parents	14 (37)
Hustling	13 (34)
Sex partner	12 (22)
Social grants	8 (21)
Friends/relatives	6 (16)
Begging	1 (3)
*multiple responses permitted; percentage is of total number of respondents.	
*Housing type,* n (%)	
Formal housing (brick house, townhouse, flat/apartment)	30 (79)
Hostel	1 (3)
Shack	7 (18)
*Perception of personal HIV risk,* n (%)	
High risk	6 (16)
Moderate risk	14 (37)
Low risk	12 (32)
No risk	5 (13)
I don’t know	1 (3)
*Perception of partner HIV risk,* n (%)	
High risk	6 (16)
Moderate risk	9 (24)
Low risk	13 (34)
No risk	3 (8)
I don’t know	7 (18)

### Main themes

Five main themes emerged.

#### Theme 1: long-lasting methods are preferable

Across all focus groups, many participants voiced a need for an HIV prevention method that requires infrequent administration, lasting for a month at least. A variety of reasons were cited: forgetfulness when using daily methods, daily methods interfered with lifestyle priorities, costliness of transportation for methods that required frequent clinic visits, and product inaccessibility because of clinic operating times.

I think if they can make it one pill for the whole month. [F 25-35]

On-demand prevention methods were perceived as impractical because the time of sexual activity was often unpredictable. Many participants perceived that barriers could potentially be offset by less frequent dosing in future innovations of long-acting methods.

Let’s say, for instance, … you didn’t take a pill because you don’t know what’s going to happen in that week. And then because of us – as the LGBTI – as we spoke earlier on, that anything (sex) can happen at any time, right? So I prefer that we, like, take an injection, … a pill in the form of injection, that will last for the whole month in your system, knowing that if anything that comes along, you know that you are already prepared in your system. [MSM 18-35]

The exception was when participants discussed a transdermal patch as a hypothetical method of HIV prevention administration: although there was little acceptability of the transdermal patch route of administration, women stated that shorter application times would be more acceptable.

#### Theme 2: condoms are well-known but not preferred for use

A prominent theme across all FGDs was that condoms are well-known to prevent HIV and STIs, but not a preferred method among participants. Most participants stated that they used condoms inconsistently. Multiple reasons for condomless sex were shared: males referred to loss of pleasure, physical and emotional intimacy, which they perceived as being derived from skin-to-skin contact during sex for both themselves and their partners.

When you are using a condom, you do not take anything that belongs to her ... But when you have unprotected sex, then that’s you and her exchanging souls. [M 25-35]

Some men stated that their choice to use condoms is influenced by an informal ‘risk assessment’ made on appearance of a sexual partner. Some women referred to condomless sex after a defined time – 3 months – in a new relationship as a sign of trust.

You being loyal to me is us sleeping without a condom. [F 18-24]

Conversely, the use of a condom was considered a form of punishment for an unfaithful partner.

#### Theme 3: administration route of HIV prevention method is a consideration for the user

All groups stated that rectal administration routes were acceptable for men who have sex with men to receive HIV prevention, but not for heterosexual populations, even though participants stated both populations can engage in anal intercourse. For heterosexual male participants, rectal administration routes of an HIV prevention method carried the stigma associated with homosexuality. MSM expressed their willingness to use rectal administration of HIV prevention, stating acceptability of rectal pills, rings and gels.

Barrier methods were perceived as theoretically ideal but stated drawbacks included discomfort, decreased sexual satisfaction and intimacy, affecting ejaculation, being oily, bad smelling, not used with regular trusted partners, and not used after the first round of sex.

Intravenous infusion methods were perceived as painful, time-consuming, and that they ought to be reserved only for hospitalised and critical care patients. Some men perceived that infusions could be unhygienic.

Injections were favoured by male and female participants and were perceived as advantageous because they were thought to work quickly systemically. There was general support for injectable PrEP innovations and for vaccine innovations. Although injections were perceived to be painful, participants were willing to use them because they were perceived to be a route of administration linked with efficacy, discreet, and long-lasting.

Many participants in all groups mistrusted the efficacy of a transdermal patch. Stated barriers were that it was perceived as an inefficacious administration route, lifestyle concerns about it possibly falling off when sweating, bathing or during rough sex, concerns about odours with extended application, and that its visibility might serve as evidence of infidelity. A woman stated:

If a patch can’t prevent small diseases like diabetes, why would it prevent a disease as big as HIV? [F 25-35]

The difficulty of swallowing large pills was mentioned as a barrier to PrEP uptake by male and female participants. One woman mentioned that she crushes large pills because she cannot swallow them whole, so a daily routine of crushing PrEP would be too laborious for her to consider PrEP uptake. Suggestions of liquid formulations commonly arose.

Additionally, the similarity of oral PrEP tablets to HIV treatment medication was mentioned as a barrier to use with participants highlighting PrEP and HIV stigma as an additional obstacle.

There is a stigma that goes with tablets with my people when people see you drinking that medication. They automatically assume that it’s ARVs (for HIV treatment). [MSM 18-35]

#### Theme 4: ideal HIV prevention methods should blend into the lifestyle of the user

Participants wanted HIV prevention strategies that could fit in with their lifestyles. One woman stated:

I want to prevent but not change my lifestyle. [F 25-35]

Participants suggested hypothetical administration methods for HIV prevention that would blend into their lifestyles: wearable devices (e.g. jewellery or clothing), food products (e.g. beverage, salt, chewing gum) and body lotion.

Some stated a preference to minimise clinic visits, suggesting multi-purpose HIV and pregnancy prevention technologies.

Most participants in all FGD groups expressed low acceptability of daily pill taking for HIV prevention. Participants raised concerns around the need for strict adherence being discordant with lifestyle: forgetting to take the pill, not mixing the pill with alcohol, and their perceived need for pills to be taken with food. A woman reported:

As much as I fear HIV with my entire life (laugh), I don’t want to live on a pill. [F 25-35]

A man stated:

Maybe you are going to a party. You cannot take the pill because you are at a party. [M 25-35]

#### Theme 5: visible prevention methods are perceived to indicate sexual indiscretion

Methods – such as tablets, transdermal patch and vaginal gels – which could be visible to others were perceived to indicate multiple partnerships and could result in mistrust, conflict and stigma, regardless of sexual orientation. Male participants repeatedly raised concerns about the visibility of transdermal patches as evidence of women’s infidelity.

It (transdermal patch) already reveals that I cheated on my partner. Don’t you think it is going to cause conflict between me and my partner?” [MSM 18–35]

Participants said that visible HIV prevention methods for women would result in male partner distrust. Heterosexual men perceived that women who were engaging in HIV prevention methods (e.g. discussing antiretroviral methods for prevention, or wearing a hypothetical transdermal patch) signified that those women had multiple sexual partners. Men perceived that it was unacceptable for their trusted main partners to use vaginal gels, because the gel would be detectable by the man, and it was perceived that trust implied that an HIV prevention method was unnecessary.

Women recommended that HIV prevention strategies – the method itself and any related adverse effects – should not be visible to male partners. A woman stated:

I am taking it (PrEP) secretly. So, what I don’t want is a situation where I become ill, you understand, and my partner will have those questions about what’s making me ill. [F 25-35]

Furthermore, the importance of discretion was underlined by societal and cultural practices which maintain a taboo around disclosing sexual behaviour and even create embarrassment, for example when accessing barrier methods from public spaces. One man stated:

Our families have got this ancient belief, ethics, ancient rules. At home they believe I am a virgin - then I come with a patch? They will chase me out of the house. [M 25-35]

## Discussion

Participants expressed a preference for long-lasting, discreet methods of HIV prevention that blend into lifestyles. Resultantly, injectable administration was perceived as acceptable, but pills and condoms were less preferred.

Other researchers – who have approached this topic through randomised crossover trial designs, discrete choice experiments and focus groups about HIV prevention method preference in Africa – have found that highly efficacious [21, 22], multi-purpose [21, 22] and long-lasting [21–23] methods were commonly the most preferred, but daily oral tablets [22] and monthly rings [22] were least preferred. Many studies in this field advocate for a variety of prevention methods suitable for different preferences.

Although multiple studies have investigated HIV prevention preferences, our study offers two main strengths. First, it provides the perspective of preference in the context of an HIV vaccine trial. This is important not only because it allows us to explore combination prevention approaches with vaccine trials. It also allows us to understand barriers beyond access, because trial sites represent idealised environments which minimise institutional barriers to access prevention knowledge and interventions. For example, the HVTN 702 trial offered pre-exposure prophylaxis at its trial sites while there was no generalised national roll out. Regardless, the trial participants in this study still cited many barriers to using HIV prevention methods that were well within their access. Second, it allowed participants the opportunity to “design their own” HIV prevention, providing useful information for novel product development and delivery mechanisms.

Our study provides candid discussions about the barriers to the two existing methods regarded as the pillars of HIV prevention, especially in at-risk populations: condoms and daily oral PrEP use. These barriers were such that some are not easily amenable to be overcome by the health education interventions recommended in standard risk reduction counselling. Although multiple HIV prevention methods are available in the public sector, their adoption by at-risk populations may not be totally self-determined, but rather influenced by partners, families, and economics amongst other factors. In South Africa, gender inequality, including power imbalances and gender-based violence, are pertinent to the question of preferred HIV prevention methods [24]. It is in this context that our participants expressed preferences for long-lasting, discreet, lifestyle-friendly methods to prevent HIV acquisition. No such method is currently on the market. Our study exposes a critical unmet need in our array of HIV prevention tools, and underscores the importance of developing biomedical innovations with greater relevance to at-risk populations, such as vaccines, long-acting passive immunisation approaches, and long-acting injectable chemoprophylaxis.

Previous experience with a prevention method may have influenced perspectives on preference. Although condom distribution programmes are well-established in South Africa, at the time of our study the PrEP programme in South Africa was in its infancy. Launched in 2016, the PrEP programme in South Africa has reached out to most-at-risk populations: sex workers, and more recently MSM, adolescent girls and young women. Thus far, uptake has been low, with one source reporting 13,500–14,500 users in the country by 01 May 2019 [25]. Although most participants in our study had basic knowledge about PrEP, acceptability was low. This is, perhaps, to be expected: possibly individuals who find daily oral tablets unacceptable are more likely to participate in a trial of an alternative method for HIV prevention. Participants in our study cited low acceptability of PrEP pills because pills were perceived as forgettable, not consistent with a lifestyle of socialising and alcohol use, too large to swallow easily, a hazard for inadvertent disclosure of risky behaviours, and stigmatised due to their similarity to HIV treatment.

The observation of herd immunity in vaccinology suggests the importance of a uniform approach to prevention, even for HIV. In one mathematical model, if prevention resources were restricted to high-prevalence locations, 41% fewer infections would be averted than if resources were available at all locations regardless of HIV prevalence [26]. The desire for participants in our study to have their HIV prevention method blend into their lifestyle is an important concept for discussion, because it prompts a conversation about how HIV prevention methods could be developed to be widely palatable for a uniform, rather than a restricted, approach. It highlights the aspiration for healthy HIV-uninfected individuals to incorporate HIV prevention into a wellness routine. In contrast, some existing HIV prevention methods are associated by participants with illness – for example antiretroviral tablets used for PrEP are primarily associated with HIV treatment [27]. The suggestions made by participants in our study for a food-like HIV prevention method provide avenues for innovation to create an aspirational health, instead of illness, platform for HIV prevention. Already, 3D printing technology science can print food [28] and is investigating the printing of medication [29] and nutraceuticals [30]. Printing HIV prevention medication into food is not a far-fetched prospect.

Our participants were all HIV vaccine trial participants who were receiving intramuscular injections as part of the trial. Therefore, a caveat in interpretation is a possible limitation in generalisability: we would expect that individuals in a vaccine trial would tend to find injectable products acceptable. Regardless, our study offers valuable insights into how patients might approach combination HIV prevention in a future context of HIV vaccine programmes, and brings to the fore the reality that at present, current prevention methodologies do not always speak to the needs of at-risk populations at the frontlines of the epidemic. A second limitation affecting generalisability is that almost all participants in this study reported being unmarried. Therefore, we are unable to offer data on union-based HIV avoidance strategies and, for example, the gender and family dynamics thereof. Within our context, the resulting bias is likely to be minor: in 2016 in South Africa, a minority (28.3%) of people aged 15 years and older were married [31]. Finally, we considered that facilitator characteristics may have introduced bias. We minimised interviewer bias towards different HIV prevention strategies by ensuring interviewers were not part of strategy-specific programmes or trials. In FGDs with women, we matched interviewer and participant gender to improve participant comfort level, and this may have introduced bias toward female-led prevention methods.

## Conclusions

Although multiple HIV prevention methods exist, participants describe seemingly incontrovertible barriers to adopting some of them, including condoms and daily oral PrEP. Participant preferences for long-lasting, discreet, lifestyle-friendly HIV prevention methods reveal an obvious gap in the biomedical prevention market aiming to reduce sexually acquired HIV. Product developers should prioritise the development of long-acting injectable formulations and vaccines, and consider novel prevention products which blend into lifestyles and promote health instead of focusing on illness.

## Data Availability

Data that support the findings of this study are available from Fatima Laher but data are not publicly available owing to the need to protect participant confidentiality.

## Abbreviations

HIV: Human Immunodeficiency Virus
VMMC: Voluntary medical male circumcision
PEP: Post-exposure prophylaxis
STIs: Sexually transmitted infections
MSM: Men who have sex with men
FGDs: Focus group discussions
PHRU: Perinatal HIV Research Unit
